# *LbKAT3* may assist in mycorrhizal potassium uptake, and overexpression of *LbKAT3* may promote potassium, phosphorus, and water transport from arbuscular mycorrhizal fungi to the host plant

**DOI:** 10.3389/fpls.2023.1161220

**Published:** 2023-06-20

**Authors:** Xia Han, Yuhao Zhou, Yanpeng Li, Wei Ren, Kunkun Liu, Wenrui Zhang, Haoqiang Zhang, Ming Tang

**Affiliations:** ^1^ College of Forestry, Northwest A&F University, Yangling, Shaanxi, China; ^2^ State Key Laboratory of Conservation and Utilization of Subtropical Agro-bioresources, Guangdong Laboratory for Lingnan Modern Agriculture, Guangdong Key Laboratory for Innovative Development and Utilization of Forest Plant Germplasm, College of Forestry and Landscape Architecture, South China Agricultural University, Guangzhou, Guangdong, China

**Keywords:** arbuscular mycorrhizal fungi, aquaporin, *Lycium barbarum*, potassium, phosphorus, tobacco

## Abstract

Potassium plays important roles in most plant physiological processes. Arbuscular mycorrhizal (AM) fungi promote plant water and mineral nutrient acquisition to promote plant growth. However, few studies have focused on the effect of AM colonization on potassium uptake by the host plant. In this study, the effects of an AM fungus (*Rhizophagus irregularis*) and potassium concentration (0, 3, or 10 mM K^+^) on *Lycium barbarum* were evaluated. A split-root test with *L. barbarum* seedlings was conducted, and the potassium uptake capacity of LbKAT3 was verified in yeast. A tobacco line overexpressing *LbKAT3* was generated and mycorrhizal functions under two potassium concentrations (0.2 and 2 mM K^+^) were studied. Inoculation of *R. irregularis* and application of potassium increased the dry weight, and potassium and phosphorus contents of *L. barbarum*, and increased the colonization rate and arbuscule abundance of *R. irregularis*. In addition, the expression of *LbKAT3* and *AQP* genes in *L. barbarum* was upregulated. Inoculation of *R. irregularis* induced *LbPT4*, *Rir-AQP1*, and *Rir-AQP2* expression, and application of potassium upregulated the expression of these genes. Inoculation with the AM fungus locally regulated the expression of *LbKAT3*. Inoculation of *R. irregularis* improved the growth, and potassium and phosphorus contents, and induced *NtPT4*, *Rir-AQP1*, and *Rir-AQP2* expression in tobacco overexpressing *LbKAT3* under both potassium concentrations. Overexpression of *LbKAT3* in tobacco improved the growth, potassium accumulation, and AM colonization, and upregulated the expression of *NtPT4* and *Rir-AQP1* in mycorrhizal tobacco. The results suggest that *LbKAT3* may assist in mycorrhizal potassium uptake, and overexpression of *LbKAT3* may promote potassium, phosphorus, and water transport from the AM fungus to tobacco.

## 1 Introduction

Potassium is among the most important nutritional elements in plants, constituting 2% to 10% of plant dry weight, and plays crucial roles in many fundamental physiological and biochemical processes (Wang and Wu, 2013). To fulfill the demand for potassium, plants have developed high-affinity transport systems by the roots or established plant–microbe associations (Garcia and Zimmermann, 2014; Liu et al., 2019).

Potassium uptake and transport within plants are mainly reliant on potassium channels and potassium transporters (Wang and Wu, 2013). When the potassium concentration in the soil is greater than 0.1 mM, plants absorb potassium through the *Shaker* family of potassium channels (Nieves-Cordones et al., 2014). Within the *Shaker* family, AKT1 plays a major role in potassium uptake and is the only potassium channel that can uptake potassium at a very low concentration (10–200 μM) (Rubio et al., 2008). SKOR controls potassium release into the xylem sap for potassium transport from the root to the shoot; AKT2 is involved in long-distance potassium transport via the phloem sap and transports potassium from the shoot to the root (Gaymard et al., 1998; Pilot et al., 2003). AtKC1 does not uptake and transport potassium on its own, but forms heteromeric channels with other members of the *Shaker* family (Jeanguenin et al., 2011). However, Sano et al. (2007) observed that, unlike AtKC1, NtKC1 has potassium uptake capacity.

Arbuscular mycorrhizal (AM) fungi, which are classified in the monophyletic division Glomeromycota, may colonize more than 80% of terrestrial plants (Smith and Smith, 2011), and are among the most important symbiotic fungi in nature (Liu et al., 2019). Compared with non-mycorrhizal plants, mycorrhizal plants absorb water and nutritional elements not only directly through roots, but also through the AM fungal mycorrhizal pathway (Javot et al., 2007; Wang et al., 2020). Highly accumulated potassium in AM fungal structures (spores, hyphae, and vesicles) and in some plant tissues implies that AM fungi facilitate potassium uptake and transport to the host plant (Garcia and Zimmermann, 2014). With consideration of the important role of phosphate transport in maintenance of the AM symbiosis (Smith et al., 2003; Javot et al., 2007), extra potassium is documented to increase phosphorus transport via the mycorrhizal pathway (Han et al., 2022b).

Aquaporins (AQPs) are responsible for water uptake and transport in plants (Moshelion et al., 2014; He et al., 2016; Hu et al., 2017a). The symbiosis with AM fungi increases water uptake and influences *AQP* expression in the host plant (Hu et al., 2017a). Moreover, the significant induction of AM fungal *AQP* expression following AM fungus inoculation suggests that host plants can directly uptake water via the mycorrhizal pathway (Porcel et al., 2006; Aroca et al., 2007). Extra potassium application upregulates the expression of *GintAQP1*, suggesting that extra potassium can increase water transport from the AM fungus to the host plant and improve the root hydraulic properties of plants (El-Mesbahi et al., 2012).

*Lycium barbarum* L. is an economically important traditional medicinal mycorrhizal plant, which is widely distributed in arid and semi-arid regions of northwestern China (Hu et al., 2017a; Zhang et al., 2017). Based on an independent *de novo* transcriptomic analysis (unpublished), the expression of a putative ortholog of *NtKC1* (designated *LbKAT3*) was significantly increased by colonization of *L. barbarum* roots by *Rhizophagus irregularis*. We speculated that *LbKAT3* may participate in potassium uptake, which may be accompanied by phosphorus and water uptake, via the mycorrhizal pathway. Therefore, the present study evaluated the effects of AM fungus inoculation and potassium application on the growth and potassium and phosphorus uptake of *L. barbarum*, the expression of *LbKAT3* and *LbPT4* in *L. barbarum*, and the expression of *AQP* genes in *L. barbarum* and *R. irregularis*. A split-root system was used to verify whether colonization by the AM fungus systemically or locally upregulated the expression of *LbKAT3*. We assessed the potassium uptake activity of LbKAT3 by means of a complementation analysis in the yeast (*Saccharomyces cerevisiae*) potassium uptake-defective mutant strain CY162. In addition, *LbKAT3* was overexpressed in tobacco, and the biomass, potassium, and phosphorus contents of the transgenic tobacco, and the expression of *LbKAT3* and *NtPT4* in tobacco and *AQP* genes in *R. irregularis* were determined.

## 2 Materials and methods

### 2.1 Plant material and growth conditions

#### 2.1.1 Experiment 1

The sterilization, germination, and cultivation of *L. barbarum* seeds followed the methods of Han et al. (2022a). At 4 weeks after germination, uniform seedlings were selected and grown in pots (10 cm × 10 cm × 9.6 cm) containing 500 g of growth substrate (soil:sand = 1:2, v/v). The soil and sand were sieved through a 2-mm-mesh sieve and sterilized before transplantation. A total of 12 treatments were applied in this experiment with three replicates per treatment. Three pots, each containing two seedlings, formed one replicate of each treatment; thus, in total, 18 plants (nine pots) were used for each treatment.

During transplantation, 15 g of inoculum of *R. irregularis* was applied beneath the seedlings as the AM treatment. The same amount of sterilized inoculum and 10 ml of filtrate (<20 mm) of the inoculum were applied for the NM treatment. After transplantation, the substrate was watered daily to keep it moist. Seedlings were fertilized every 10 days with 15 ml of Hoagland’s solution containing 10% phosphate (0.1 mM KH_2_PO_4_). At 100 days after transplantation, three concentrations of K_2_SO_4_ solution were applied to the relevant pots every 2 days for a total of three applications (20 ml per application; a total of 60 ml was applied to achieve final concentrations of 0, 3, and 10 mmol K^+^/kg growth substrate, respectively designated K1, K2, and K3 treatments), and lasted for 50 days after application of potassium.

#### 2.1.2 Experiment 2

Uniform *L. barbarum* seedlings were selected and grown using a split-root system (**Supplementary Figure 1**). The AM and NM treatments were applied in this experiment. *R. irregularis* inoculum (10 g) was applied beneath the seedlings in the AM root compartment during transplantation, and sterilized 10 g of inoculum (121°C for 2 h in an autoclave) and 10 ml of filtrate (<20 mm) of the AM inoculum were applied in the non-mycorrhizal root compartment of the AM treatment and in the NM treatment. After transplantation, the substrate was kept moist by watering daily and was fertilized weekly with 20 ml of Hoagland’s solution containing 10% phosphate (0.1 mM KH_2_PO_4_). After growth for 6 weeks, the seedlings were harvested. Three roots in the same root compartment of each treatment formed one replicate and, in total, three replicates were included for each root compartment.

#### 2.1.3 Experiment 3

Uniform tobacco seedlings of the two lines (wild-type *N. tabacum*, WT; overexpression of LbKAT3, OE) cultured in tissue culture flasks were selected for transplantation. The seedlings were grown in pots (10 cm × 10 cm × 9.6 cm) containing 400 g of sterilized growth substrate (vermiculite:sand = 1:1, v/v). The AM and NM treatments were applied as described for Experiment 1. After transplantation, the substrate was watered daily and was fertilized weekly with 20 ml of Hoagland’s solution containing 10% phosphate (0.1 mM KH_2_PO_4_) to ensure a high frequency of mycorrhizal colonization. After 4 weeks of tobacco growth, two concentrations of K_2_SO_4_ solution (20 ml) were applied to the corresponding pots (0.2 and 2 mmol K^+^/kg growth substrate in the corresponding pots) and growth continued for an additional 2 weeks. There were four replicates for each treatment (eight in total), and 32 pots in total, in this experiment.

### 2.2 Plant growth and mycorrhizal colonization

At harvest, the shoots and roots were separated. The fresh shoot and root weights of each replicate were recorded. One portion of the roots was cut into 1-cm-long fragments and stained with trypan blue (Koske and Gemma, 1989). The AM colonization rate was determined using the magnified intersection method under a light microscope (Mcgonigle et al., 1990). Portions of the shoots and roots were dried at 65°C until constant weight and used for measurement of nutrient contents. The remaining portions of shoots and roots were stored at −80°C after snap-freezing in liquid nitrogen.

### 2.3 Potassium and phosphorus contents

The dried plant material was digested as described in a previous study (Han et al., 2022a). The potassium concentration was measured using a flame atomic absorption spectrometer (PinAAcle 500, PerkinElmer, Inc., Shelton, CT, USA). The phosphorus concentration was measured using the molybdenum yellow colorimetric method (Page, 1982). The contents of potassium and phosphorus were calculated from the respective concentration and dry weight.

### 2.4 Isolation and computational analysis of putative gene *LbKAT3*

Based on independent *de novo* transcriptome sequencing, a partial sequence for *LbKAT3* was obtained. The 5′ and 3′ rapid amplification of cDNA ends (RACE) procedure (Bertioli, 1997) was performed using the SMARTer™ RACE cDNA Amplification Kit (Clontech Laboratories, Inc., Mountain View, CA, USA) to amplify the full-length sequence for *LbKAT3*. The primers used are listed in **Supplementary Table 1**.

The Open Reading Frame Finder tool of the NCBI (https://www.ncbi.nlm.nih.gov/orf finder/) was used to analyze the open reading frame (ORF) and to predict the amino acid sequence. The molecular weight and isoelectric point of the protein was predicted by the ProtParam tool (http://web.expasy.org/protparam/). The subcellular localization of the protein was predicted using the TargetP 2.0 Server (http://www.cbs.dtu.dk/services/TargetP/). Transmembrane helices in the protein were predicted by Deep TMHMM (https://dtu.biolib.com/DeepTMHMM). A neighbor-joining tree was constructed using MEGA 6.06.

### 2.5 Relative gene expression in *L. barbarum* and tobacco roots, and in *R. irregularis*

Total RNA was extracted from 100 mg of powdered roots, from material stored at −80°C, using the E.Z.N.A.™ Plant RNA Kit (Omega Bio-Tek, Norcross, GA, USA). The first-strand cDNA synthesis was obtained from 1 μg of good-quality total RNA using the TIANScript RT Kit (TIANGEN Bio, Beijing, China) following the supplier’s instructions. The cDNA was used as the template for PCR reactions.

Quantitative real-time PCR (qRT-PCR) reactions were performed following the method of Han et al. (2022a) using the SYBR Green Master Mix (Roche Diagnostics, Basel, Switzerland). The gene-specific primers used are listed in **Supplementary Table 1**. The *L. barbarum Actin* gene and tobacco and *R. irregularis Elongation Factor 1-α* genes were used as internal controls to estimate the relative transcript abundance of target genes in *L. barbarum* and tobacco roots and in *R. irregularis*, respectively. The relative expression was calculated as 2^−Δ^ *^C^* ^t^ (where Δ*C*_t_ = *C*_t_[gene of interest] − *C*_t_[internal control]).

### 2.6 Construction of a binary vector and plant transformation

The full-length coding sequence of *LbKAT3* was inserted into the plant expression vector pROKII using the ClonExpress II One Step Cloning Kit (Vazyme, Nanjing, China). The recombinant vector, named *LbKAT3-pROKII*, was transformed into *Agrobacterium tumefaciens* strain GV3101 for transformation of tobacco. *Agrobacterium tumefaciens*-mediated transformation of tobacco was conducted using the leaf disc method and the transformants were grown on Murashige and Skoog medium (Cheng et al., 2022). The transcript abundance of *LbKAT3* in the overexpression line was confirmed by qRT-PCR (**Supplementary Figure 2**).

### 2.7 Functional characterization of *LbKAT3* in yeast

The full-length coding sequence of *LbKAT3* was inserted into the yeast expression vector pYES2. The resulting vector was transformed into the yeast potassium uptake-defective mutant strain CY162 (MATα ura3-52 his4-15 trk1Δ trk2Δ1::pCK64), which was provided by the National BioResource Project (NBRP) of the Ministry of Education, Culture, Sports, Science and Technology (MEXT), Japan. The wild-type yeast strain BY4741 was donated by Professor Chen Peng (College of Life Sciences, Northwest A&F University). Yeast cell transformation was performed using the LiAC/single-stranded DNA/polyethylene glycol method, and growth was assayed on arginine phosphate (AP) medium (supplemented with galactose) (Horie et al., 2011).

### 2.8 Statistical analysis

Statistical analysis was performed using IBM SPSS Statistics 21.0 software (IBM, Armonk, NY, USA). The significance of differences among experimental groups was assessed using Duncan’s multiple range test. Correlation analyses were performed using Spearman’s rank correlation test. Figures were generated with Origin Pro 2018 (Origin Lab, Northampton, MA, USA).

## 3 Results

### 3.1 Identification of *LbKAT3* in *L. barbarum* and functional analysis of *LbKAT3* in yeast

**Figure 1:**
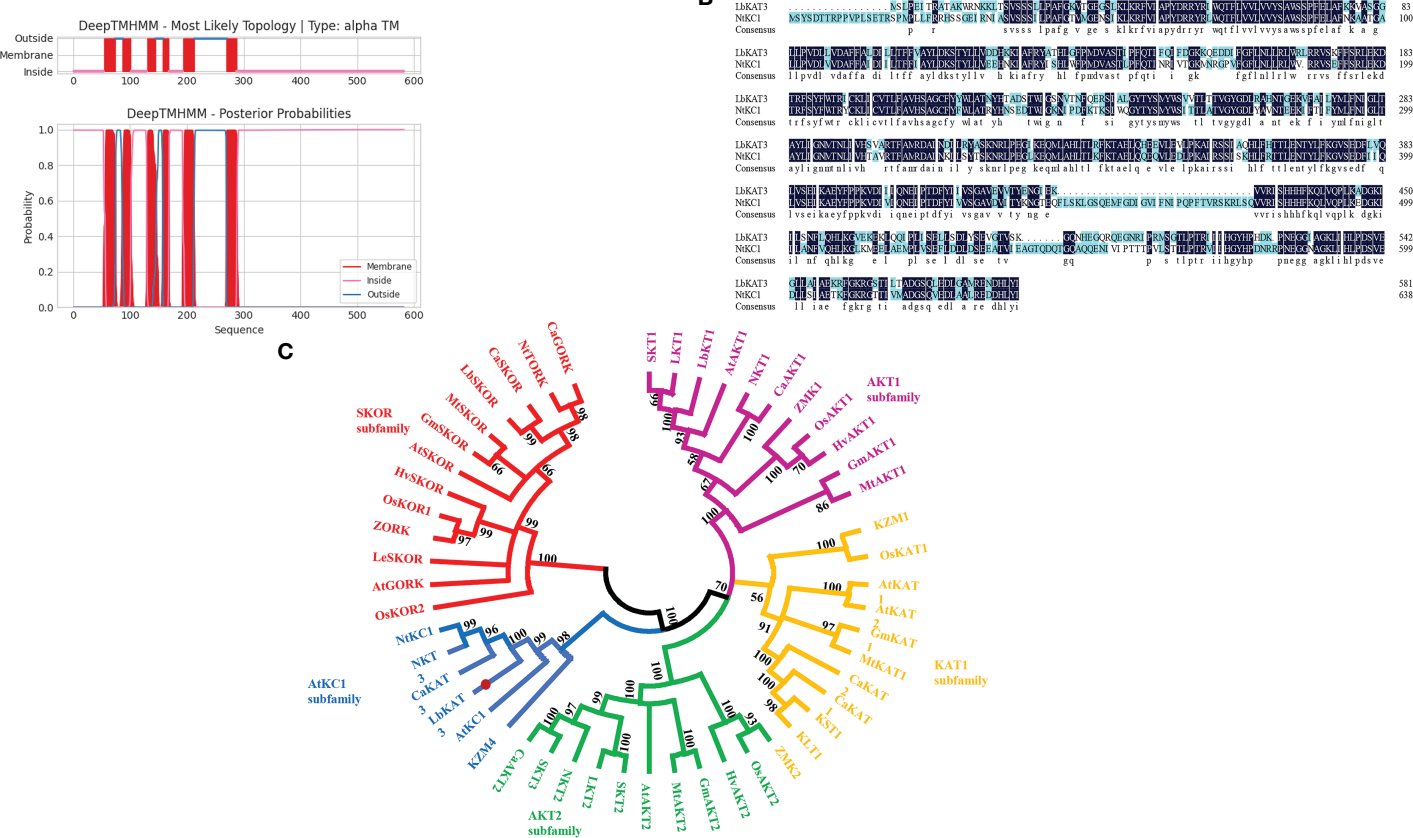
Putative transmembrane domains of LbKAT3 **(A)**, multiple alignment of the deduced amino acid sequences of LbKAT3 in *Lycium barbarum* with NtKC1 in tobacco **(B)**, and neighbor-joining tree for plant potassium channels **(C)**.

A full-length cDNA encoding a potassium channel from the *Shaker* family was obtained, named *LbKAT3*, and deposited in GenBank (accession MZ416923.1). The ORF of *LbKAT3* was 1,749 bp, which encoded 582 amino acids, and the predicted molecular weight of LbKAT3 was 66.26 kDa. The putative isoelectric point of LbKAT3 was 9.00 and the protein was predicted to be localized to the plasma membrane (**Table 1**). Analysis of the deduced protein revealed that LbKAT3 was predicted to have six transmembrane helices (**Figure 1A**). Based on a multiple sequence alignment and construction of a neighbor-joining tree, LbKAT3 was indicated to belong to the AtKC1 subfamily of the *Shaker* family and was an ortholog of NtKC1 (**Figures 1B, C**).

**Table 1:** Prediction of physiochemical properties and subcellular localization of LbHAK and LbKAT3.

Gene name	GenBank accession	ORF length (bp)	Protein length (aa)	Molecular weight (kDa)	Isoelectric point	Subcellular location
LbKAT3	MZ416923	1,749	582	66.26	9.00	Plasma membrane

**Figure 2:**
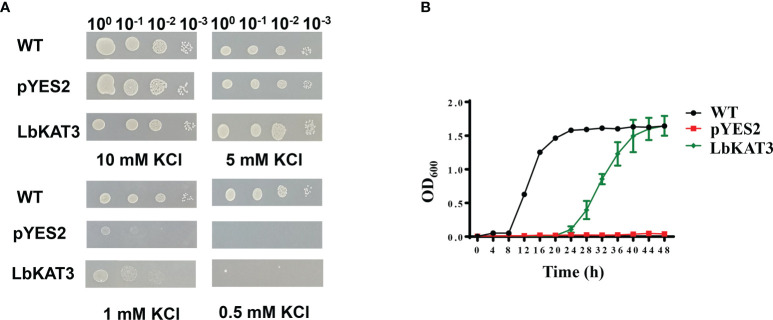
Functional complementation of LbKAT3 for K^+^ acquisition in the yeast mutant strain CY162. **(A)** Growth status of CY162 cells expressing LbKAT3 and empty vector (pYES2), and the wild type (WT; strain BY4741) on AP medium supplemented with 10 mM, 5 mM, 1 mM, or 0.5 mM KCl. **(B)** Growth curve of the yeast CY162 cells expressing LbKAT3 and empty vector (pYES2), and WT (BY4741) cultured in liquid AP medium containing 0.5 mM KCl. Data are presented as the mean ± SD (*n* = 3).

The yeast potassium uptake-defective mutant CY162 was used for complementation analysis on AP medium containing different concentrations of potassium. All tested yeast strains grew well under the 5 and 10 mM potassium concentrations. Under the 1 mM potassium concentration, the growth of CY162 yeast harboring pYES2 and *LbKAT3* was inhibited. With the reduction in the potassium concentration to 0.5 mM, the growth of the CY162 yeast harboring pYES2 was entirely suppressed, whereas growth of the CY162 yeast containing *LbKAT3* was largely inhibited but still showed weak growth (**Figure 2A**). Growth curve showed that in liquid AP medium containing 0.5 mM KCl, the yeast expressing *LbKAT3* and the WT grew much faster than the yeast harboring the empty vector, and the WT yeast grew faster than the yeast expressing *LbKAT3* (**Figure 2B**), suggesting that *LbKAT3* has potassium uptake activity, but it is not especially strong. The difference in results for the CY162 yeast harboring *LbKAT3* between the drop test assays and the growth curve might be due to the addition of 75-times more bacterial liquid in the growth curve than in the drop test assays.

### 3.2 AM fungus and extra potassium improve growth and potassium and phosphorus uptake of *L. barbarum*

Inoculation of *R. irregularis* significantly improved both shoot and root growth of *L. barbarum* (**Table 2**). Application of potassium significantly improved the growth of mycorrhizal seedlings, and the K2 treatment significantly increased the root biomass of non-mycorrhizal plants (**Table 2**). These results showed that inoculation of *R. irregularis* had a stronger influence on *L. barbarum* growth than that of potassium application, and that the treatments had synergistic effects on the growth of *L. barbarum*.

**Table 2:** Biomass and AM colonization rates of *L. barbarum* under different treatments.

Treatment	Dry weight of shoot (g)	Dry weight of root (g)	Colonization rate of AM fungi (%)	Arbuscule abundance (%)
NMK0	0.17 ± 0.01 d	0.22 ± 0.01 d	ND	ND
NMK1	0.24 ± 0.04 d	0.29 ± 0.05 cd	ND	ND
NMK2	0.26 ± 0.03 d	0.32 ± 0.01 c	ND	ND
AMK0	1.13 ± 0.18 b	0.80 ± 0.03 b	60.54 ± 5.12 b	16.97 ± 1.02 c
AMK1	1.34 ± 0.11 b	1.00 ± 0.08 a	69.20 ± 3.73 a	21.65 ± 1.04 b
AMK2	1.44 ± 0.15 a	1.01 ± 0.03 a	73.82 ± 1.92 a	24.45 ± 1.75 a
*P_AMF_*	**	***	NA	NA
*P_k_*	*	***	*	**
P_AMF*K_	NS	*	NA	NA

NM, non-mycorrhizal; AM, inoculated with R. irregularis; K0, K1, and K2, 0 mmol, 3 mmol, and 10 mmol K^+^ (K_2_SO_4_)/kg substrate, respectively; NS, not significant; NA, not applicable; ND, not detected. Data are presented as the mean ± SD; * represents *p* < 0.05; ** represents *p* < 0.01; *** represents *p* < 0.001. Different letters indicate that the means are significantly different between treatments. Analysis of variance followed by Duncan test when the whole test was significantly different (*p* < 0.05); n = 3.

No mycorrhizal colonization was observed in non-mycorrhizal plants. Application of potassium significantly increased AM colonization rate and arbuscule abundance (**Table 2**), which indicated that application of potassium had a positive effect on mycorrhizal colonization.

The potassium content in the shoot was higher than that in the root (**Table 3**). Inoculation of *R. irregularis* increased the shoot and total potassium contents under all potassium concentrations, and root potassium content under the K1 and K2 treatments (**Table 3**). Application of potassium improved the shoot, root, and total potassium contents of mycorrhizal plants, and only the K2 treatment significantly increased the shoot and total potassium contents of non-mycorrhizal plants (**Table 3**). Thus, inoculation of *R. irregularis* and application of potassium had synergistic effects on potassium uptake by *L. barbarum*.

**Table 3:** Potassium and phosphorus contents of *L. barbarum* roots and leaves under different treatments.

Treatment	Potassium content (mg)			Phosphorus content (mg)		
	Shoot	Root	Total	Shoot	Root	Total
NMK0	1.91 ± 0.08 e	1.59 ± 0.18 b	3.49 ± 0.24 e	0.11 ± 0.03 d	0.04 ± 0.004 c	0.14 ± 0.04 e
NMK1	3.18 ± 0.27 e	1.83 ± 0.25 b	5.01 ± 0.52 e	0.17 ± 0.07 d	0.27 ± 0.10 bc	0.46 ± 0.18 de
NMK2	6.36 ± 2.09 d	2.49 ± 0.10 b	8.85 ± 2.00 d	0.25 ± 0.07 cd	0.29 ± 0.02 bc	0.55 ± 0.09 d
AMK0	9.32 ± 2.00 c	3.81 ± 1.82 b	13.13 ± 2.03 c	0.48 ± 0.04 c	0.45 ± 0.02 ab	0.93 ± 0.04 c
AMK1	13.27 ± 1.43 b	7.06 ± 2.43 a	20.28 ± 2.27 b	0.79 ± 0.25 b	0.62 ± 0.08 a	1.41 ± 0.33 b
AMK2	18.63 ± 1.79 a	8.61 ± 2.02 a	27.24 ± 3.45 a	1.14 ± 0.14 a	0.75 ± 0.17 a	1.89 ± 0.32 a
*P_AMF_*	***	***	***	***	***	***
*P_k_*	***	*	***	**	*	***
P_AMF*K_	*	NS	*	*	NS	NS

NM, non-mycorrhizal; AM, inoculated with R. irregularis; K0, K1, and K2, 0, 3, and 10 mmol K^+^ (K_2_SO_4_)/kg substrate, respectively; NS, not significant; NA, not applicable. Data are presented as the mean ± SD; * represents *p* < 0.05; ** represents *p* < 0.01; *** represents *p* < 0.001. Different letters indicate that the means are significantly different between treatments. Analysis of variance followed by Duncan test when the whole test was significantly different (*p* < 0.05); n = 3.

Inoculation of *R. irregularis* increased the shoot and root phosphorus contents under all potassium concentrations, and total phosphorus content under the K1 and K2 treatments (**Table 3**). Application of potassium significantly increased the shoot and total phosphorus contents of mycorrhizal plants, and K2 treatment also significantly increased the total phosphorus content of non-mycorrhizal plants (**Table 3**). These results showed that application of potassium had a positive effect on the phosphorus uptake of mycorrhizal *L. barbarum*.

### 3.3 AM fungi and extra potassium increased expression of genes associated with potassium and phosphorus uptake and transport, and regulated *AQP* expression in roots of *L. barbarum*

**Figure 3:**
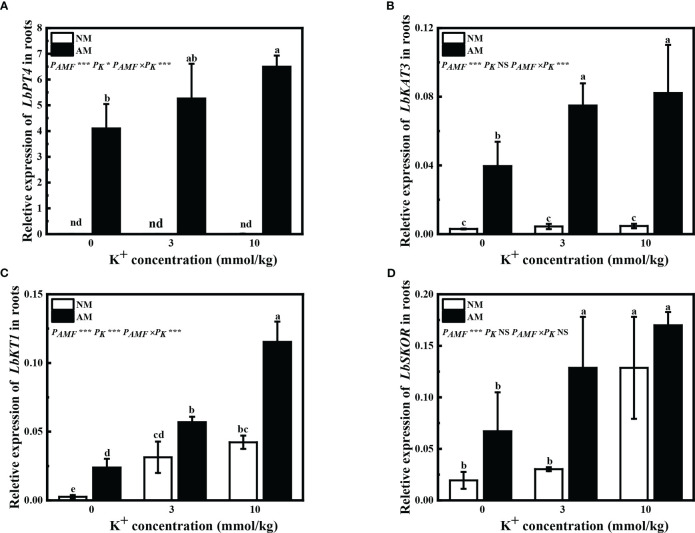
Expression of **(A)** *LbPT4*, **(B)** *LbKAT3*, **(C)** *LbKT1*, and **(D)** *LbSKOR* in roots of *Lycium barbarum* under different treatments. NM, non-mycorrhizal; AM, inoculated with *Rhizophagus irregularis*; NS, non-significant. Different letters above bars indicate a significant difference (Duncan’s multiple range test, *n* = 3). *p < 0.05; **p < 0.01; ***p < 0.001.

Expression of *LbPT4* was not detected in non-mycorrhizal plants. In mycorrhizal plants, application of 10 mM potassium significantly improved the relative expression of *LbPT4* (**Figure 3A**). The relative expression of *LbKAT3* was significantly upregulated in mycorrhizal *L. barbarum* roots (**Figure 3B**). Application of potassium significantly improved the *bKAT3* relative expression of mycorrhizal plants (**Figure 3B**). Inoculation of *R. irregularis* significantly improved the *LbKT1* relative expression, and *LbSKOR* relative expression was enhanced with extra potassium application (**Figures 3C, D**). Application of extra potassium significantly upregulated *LbKT1* relative expression by all plants and *LbSKOR* relative expression by mycorrhizal plants, and application of 10 mM potassium increased *LbSKOR* relative expression by non-mycorrhizal plants (**Figures 3C, D**). These results showed that inoculation of *R. irregularis* and application of potassium had synergistic effects on the expression of genes associated with potassium and phosphorus uptake and transport in *L. barbarum*.

**Figure 4:**
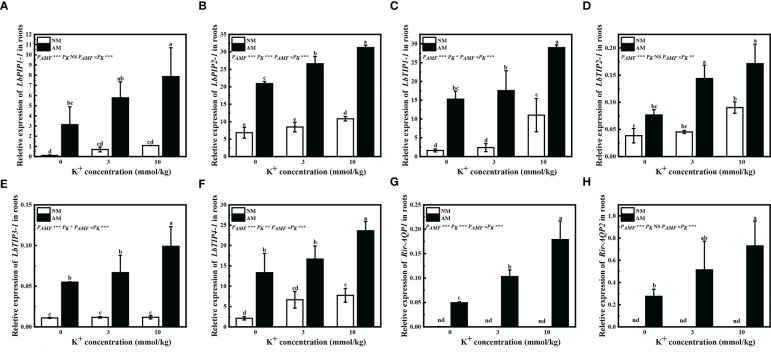
Expression of *LbPIP1-1* **(A)**, *LbPIP2-1* **(B)**, *LbTIP1-1* **(C)**, *LbTIP2-1* **(D)**, *LbTIP3-1* **(E)**, *LbTIP4-1* **(F)**, *Rir-AQP1* **(G)**, and *Rir-AQP2* **(H)** in roots of Lycium barbarum under different treatments. NM, non-mycorrhizal; AM, inoculated with Rhizophagus irregularis; NS, non-significant. Different letters above bars indicate a significant difference (Duncan’s multiple range test, n = 3). *p < 0.05; **p < 0.01; ***p < 0.001.

Inoculation of *R. irregularis* significantly increased *LbPIP1-1*, *LbPIP2-1*, *LbTIP1-1*, *LbTIP3-1*, and *LbTIP4-1* relative expression under all potassium concentrations, and *LbTIP2-1* relative expression with extra potassium application (**Figures 4A–F**). Application of potassium had little influence on *LbPIP1-1* and *LbTIP3-1* relative expression by non-mycorrhizal plants, and significantly upregulated *LbPIP2-1* and *LbTIP2-1* relative expression by mycorrhizal plants; application of 10 mM potassium significantly upregulated *LbPIP1-1*, *LbTIP1-1*, *LbTIP3-1*, and *LbTIP4-1* relative expression by mycorrhizal plants, and *LbPIP2-1*, *LbTIP1-1*, *LbTIP2-1*, and *LbTIP4-1* relative expression by non-mycorrhizal plants (**Figures 4A–F**). These results showed that inoculation of *R. irregularis* and application of potassium had synergistic effects on the expression of *AQP* genes of *L. barbarum*.

Expression of the AQP genes *Rir-AQP1* and *Rir-AQP2* was not detected in non-mycorrhizal plants (**Figures 4G, H**). Application of extra potassium significantly improved the relative expression of *Rir-AQP1* in mycorrhizal plants, and only 10 mM extra potassium significantly upregulated *Rir-AQP2* relative expression in mycorrhizal plants (**Figures 4G, H**).

### 3.4 AM fungi locally upregulated *LbKAT3* expression in *L. barbarum*

**Figure 5:**
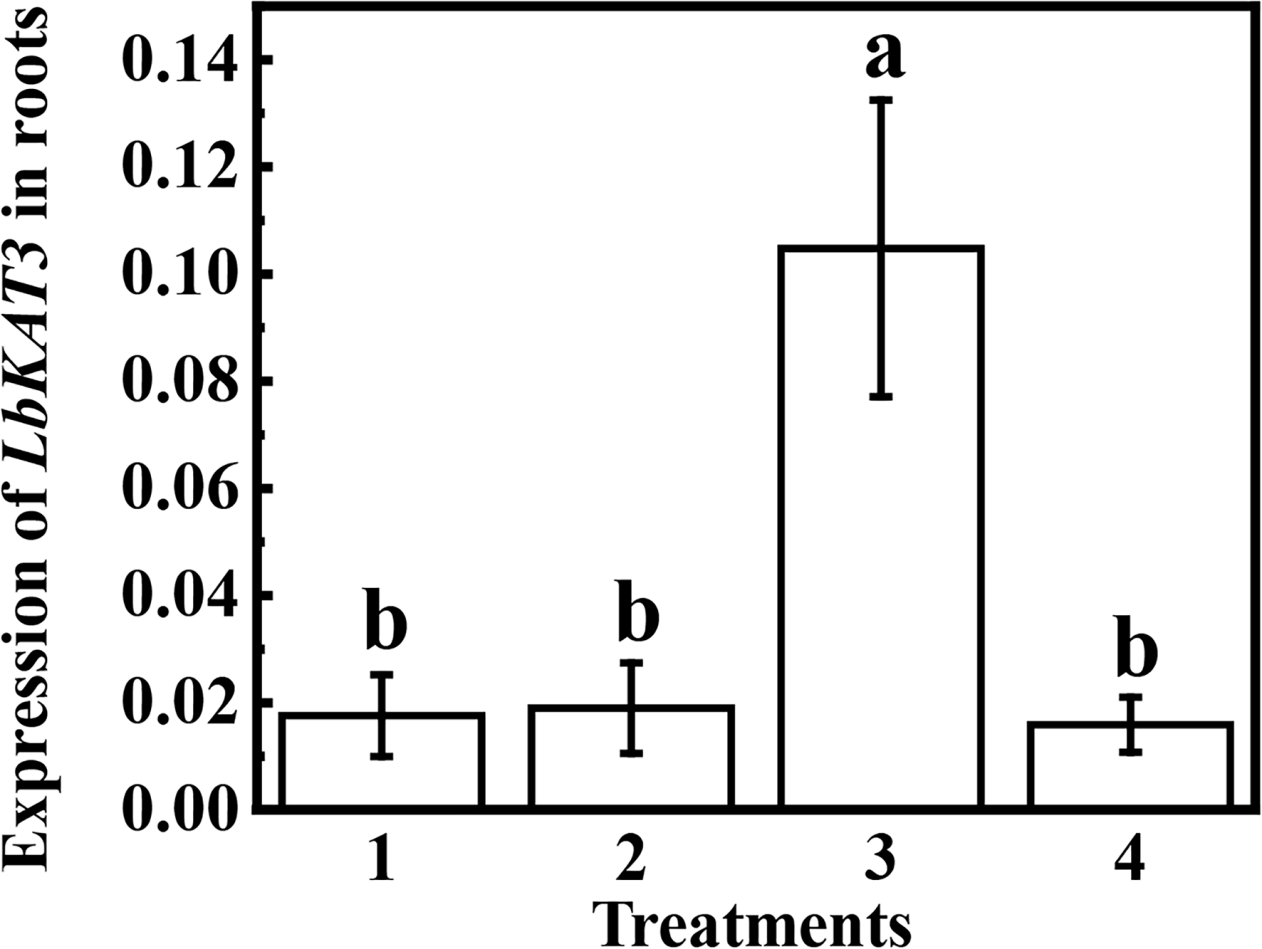
Expression of *LbKAT3* in roots of *Lycium barbarum* growing using a split-root system. 1 and 2 are the two root compartments without arbuscular mycorrhizal (AM) inoculation, 3 is the root compartment inoculated with *Rhizophagus irregularis* in the AM treatment, and 4 is the root compartment not inoculated with *R. irregularis* in the AM treatment.

Compared with non-mycorrhizal plants, inoculation of *R. irregularis* in the AM compartment of the AM treatment significantly upregulated the expression of *LbKAT3*, whereas the *LbKAT3* expression in the NM compartment of the AM treatment was similar to that of the non-mycorrhizal plants (**Figure 5**). These results indicated that the AM fungus locally upregulated the expression of *LbKAT3*.

### 3.5 Overexpression of *LbKAT3* in tobacco improved tobacco growth and mycorrhizal colonization

**Figure 6:**
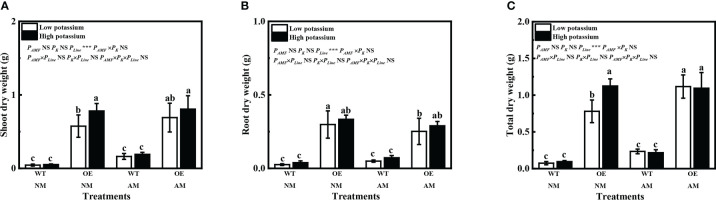
Effects of overexpression of *LbKAT3* on tobacco growth. **(A)**, shoot dry weight; **(B)**, root dry weight; **(C)**, total dry weight. Low potassium = 0.2 mmol/kg substrate; High potassium = 2 mmol/kg substrate; WT, wildtype tobacco; OE, overexpression of LbKAT3 in tobacco; NM, non-mycorrhizal; AM, inoculated with Rhizophagus irregularis; NS, non-significant. Different letters above bars indicate a significant difference (Duncan’s multiple range test, n = 4). *p < 0.05; **p < 0.01; ***p < 0.001.

The line number 10 tobacco of overexpression of *LbKAT3* was used for follow-up tests (**Supplementary Figure 2**). Overexpression of *LbKAT3* in tobacco significantly increased the tobacco shoot, root, and total dry weights (**Figures 6A–C**). Inoculation of *R. irregularis* significantly increased the total dry weight of the OE line under a low (0.2 mM) potassium concentration (**Figures 6A–C**). Application of a high (2 mM) potassium concentration only increased the growth of non-mycorrhizal plants of the OE line (**Figures 6A–C**). With regard to the WT plants, inoculation of *R. irregularis* significantly increased tobacco growth and 2 mM potassium also significantly increased the root dry weight of mycorrhizal plants (**Supplementary Figure 3**). Compared with the WT plants, inoculation of *R. irregularis* and application of 2 mM potassium had no synergistic effect on growth of the tobacco OE line, which might be due to the strong positive effect of overexpression of *LbKAT3* on tobacco growth.

**Figure 7:**
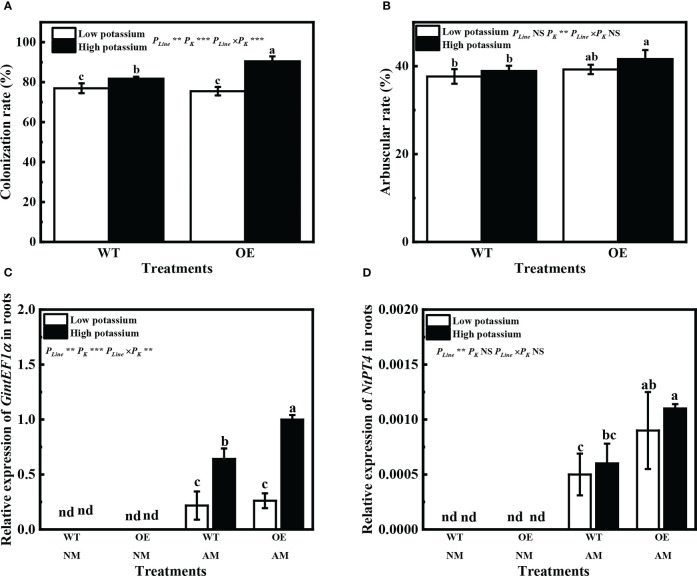
Effects of overexpression of *LbKAT3* on arbuscular mycorrhizal (AM) colonization efficiency in tobacco. **(A)** AM colonization rate; **(B)** arbuscular rate; **(C**, **D)** *GintEF1α* and *NtPT4* relative expression in mycorrhizal tobacco. Low potassium = 0.2 mmol/kg substrate; High potassium = 2 mmol/kg substrate; WT, wild-type tobacco; OE, overexpression of *LbKAT3* in tobacco; NM, non-mycorrhizal; AM, inoculated with *Rhizophagus irregularis*; NS, non-significant. Different letters above bars indicate a significant difference (Duncan’s multiple range test, *n* = 4). *p < 0.05; **p < 0.01; ***p < 0.001.

Overexpression of *LbKAT3* significantly increased the tobacco mycorrhizal colonization rate and arbuscular rate with a high potassium concentration (**Figures 7A, B**). Expression of *GintEF1α*, as a *R. irregularis* indicator gene, was used to further verify the rate of mycorrhizal colonization. The expression pattern of *GintEF1α* was consistent with the mycorrhizal colonization rate (**Figure 7C**). The tobacco gene *NtPT4* was used as a mycorrhiza-specific induced phosphate transporter gene and as an indicator gene for the arbuscular rate; its expression was only detected in mycorrhizal tobacco. The expression of *NtPT4* in mycorrhizal tobacco was significantly increased by the overexpression of *LbKAT3* under both low and high potassium concentrations (**Figure 7D**). The difference between the expression of *NtPT4* and the arbuscular rate may be due to error in visual inspection of the arbuscular rate.

### 3.6 Overexpression of *LbKAT3* in tobacco improved tobacco potassium and phosphorus uptake and the expression of associated genes

**Figure 8:**
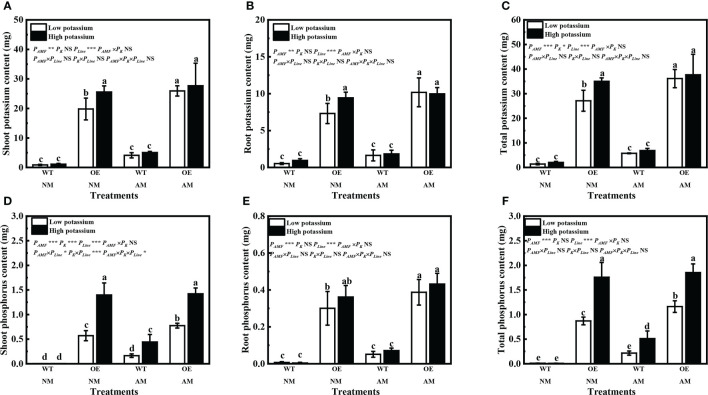
Effects of overexpression of *LbKAT3* on potassium contents (**A**, shoot; **B**, root; **C**, total) and phosphorus contents (**D**, shoot; **E**, root; **F**, total) in tobacco. Low potassium = 0.2 mmol/kg substrate; High potassium = 2 mmol/kg substrate; WT, wild-type tobacco; OE, overexpression of *LbKAT3* in tobacco; NM, non-mycorrhizal; AM, inoculated with *Rhizophagus irregularis*; NS, non-significant. Different letters above bars indicate a significant difference (Duncan’s multiple range test, *n* = 4). *p < 0.05; **p < 0.01; ***p < 0.001.

Overexpression of *LbKAT3* significantly increased the shoot, root, and total contents of both potassium and phosphorus in tobacco (**Figures 8A–F**). Inoculation of *R. irregularis* significantly increased the shoot, root, and total contents of both potassium and phosphorus of OE tobacco plants under the low potassium concentration (**Figures 8A–F**). Application of 2 mM potassium only significantly increased the shoot, root, and total contents of potassium of non-mycorrhizal OE tobacco (**Figures 8D–F**), and significantly increased the shoot and total contents of phosphorus of OE tobacco and mycorrhizal WT tobacco (**Figures 8A–C**, **Supplementary Figure 4**).

**Figure 9:**
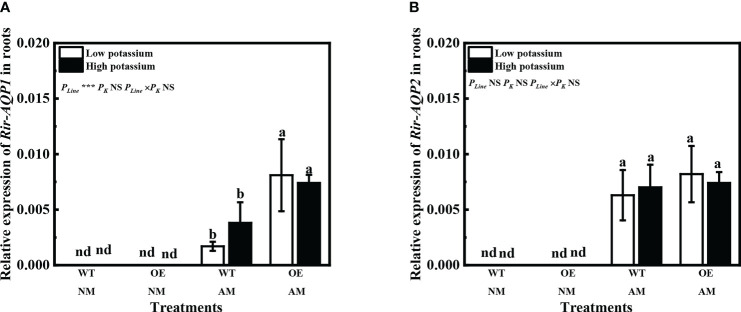
Effects of overexpression of LbKAT3 on expression of Rir-AQP1 **(A)**, and Rir-AQP2 **(B)** in tobacco. Low potassium = 0.2 mmol/kg substrate; High potassium = 2 mmol/kg substrate; WT, wild-type tobacco; OE, overexpression of LbKAT3 in tobacco; NM, non-mycorrhizal; AM, inoculated with Rhizophagus irregularis; NS, non-significant. Different letters above bars indicate a significant difference (Duncan’s multiple range test, n = 4). *p < 0.05; **p < 0.01; ***p < 0.001.

Expression of *Rir-AQP1* and *Rir-AQP2* was not detected in non-mycorrhizal tobacco (**Figures 9A, B**). Overexpression of *LbKAT3* significantly increased the relative expression of *Rir-AQP1* under both low and high potassium concentrations, but had little influence on the relative expression of *Rir-AQP2* (**Figures 9A, B**).

### 3.7 Correlation analysis

In Experiment 1, the relative expression of *LbPT4* was positively correlated with the relative expression of *LbKAT3*, *Rir-AQP1*, and *Rir-AQP2*, and with root potassium and phosphorus contents. Potassium content was positively correlated with the relative expression of *LbKAT3, Rir-AQP1*, and *Rir-AQP2*, and with phosphorus content (**Table 4**).

**Table 4:** Correlation coefficients of potassium and phosphorus concentrations, and gene expression in *L. barbarum* roots and *Rhizophagus irregularis.*

	Expression of *LbPT4*	Root potassium content	Root phosphorus content
Expression of *Rir-AQP1*	0.888^***^	0.934^***^	0.699^**^
Expression of *Rir-AQP2*	0.867^***^	0.846^***^	0.745^**^
Expression of *LbPT4*	1	0.767^***^	0.788^***^
Root potassium content	0.767^***^	1	0.665^**^
Root phosphorus content	0.788^***^	0.665^**^	1

* indicated significance of correlation coefficient at *p* < 0.05. ** indicated significance of correlation coefficient at *p* < 0.01. *** indicated significance of correlation coefficient at *p* < 0.001.

In Experiment 3, the relative expression of *NtPT4* was positively correlated with the relative expression of *Rir-AQP1*, arbuscular rate, and root potassium and phosphorus contents. Potassium content was positively correlated with the relative expression of *Rir-AQP1*, arbuscular rate, and root phosphorus content (**Table 5**).

**Table 5:** Correlation coefficients of potassium and phosphorus contents, colonization rate, and gene expression in tobacco roots and *Rhizophagus irregularis.*

	Expression of *NtPT4*	Root potassium content	Root phosphorus content
Expression of *Rir-AQP1*	0.760^**^	0.850^***^	0.826^***^
Expression of *NtPT4*	1	0.732^**^	0.720^**^
Arbuscular rate	0.552^*^	0.536^*^	0.574^*^
Root potassium content	0.732^**^	1	0.948^***^
Root phosphorus content	0.720^**^	0.948^***^	1

* indicated significance of correlation coefficient at *p* < 0.05. ** indicated significance of correlation coefficient at *p* < 0.01. *** indicated significance of correlation coefficient at *p* < 0.001.

## 4 Discussion

### 4.1 AM fungi and extra potassium improve plant growth and potassium uptake

Improvement of plant nutrient and water uptake and transport is an important function of the AM symbiosis (Chitarra et al., 2016; Ruiz-Lozano et al., 2016; Liu et al., 2019). However, the influence of the AM symbiosis on plant potassium accumulation has received much less attention than that on phosphorus and nitrogen accumulation, and might be due to the strong regulation of the potassium concentration by the plant (Wang and Wu, 2013). Inoculation of *R. irregularis* and application of potassium increased *L. barbarum* growth (**Table 1**) and potassium contents (**Table 2**), which was consistent with previous studies (Liu et al., 2019; Han et al., 2022a; Han et al., 2022b). These results highlight the positive roles of AM fungi and extra potassium in plant growth and potassium accumulation (Garcia et al., 2017; Liu et al., 2019; Han et al., 2022b). The sulfur in K_2_SO_4_ may have little influence on plant growth, because application of sulfur and inoculation of AM fungi have little influence on the expression level of a sulfate transporter in plants under the sulfur-sufficient condition (El-Mesbahi et al., 2012; Giovannetti et al., 2014). Plant potassium uptake and transport mainly depend on potassium channels and transporters (Wang and Wu, 2013). Inoculation of *R. irregularis* and application of potassium increased the relative expression of *LbKT1* and *LbSKOR* in roots of *L. barbarum*, which further indicated that AM fungi and extra potassium can increase potassium uptake and transport (Zhang et al., 2017; Han et al., 2022a; Han et al., 2022b).

The relative expression of *LbKAT3* was upregulated by inoculation of *R. irregularis* and application of potassium (**Figure 3B**), and was positively correlated with root potassium content (**Table 4**). In addition, LbKAT3, similar to its ortholog NtKC1 (Sano et al., 2007), provided capacity for potassium uptake (**Figure 2**). *LbKAT3* was highly expressed in AM fungus-colonized roots (**Figure 5**). Thus, the AM fungus locally upregulated the expression of *LbKAT3*, indicating that *LbKAT3* may assist in potassium uptake via the mycorrhizal pathway. Overexpression of *LbKAT3* significantly increased mycorrhizal tobacco biomass (**Figure 6**) and potassium contents (**Figures 8A–C**), suggesting that overexpression of *LbKAT3* increased potassium uptake and growth of tobacco, and that *LbKAT3* may assist in mycorrhizal potassium uptake to promote tobacco growth. Liu et al. (2019) reported that the AM-induced potassium transporter SlHAK10 participates in potassium transport via the mycorrhizal pathway under potassium deficiency. Hence, the increase in potassium content in tobacco may also be associated with a potassium transporter, which requires clarification by conducting further analyses.

### 4.2 Application of extra potassium improves AM colonization rate and arbuscular rate

AM fungi can colonize more than 80% of vascular plants to promote plant nutrient and water uptake, enhance resistance to biotic and abiotic stresses, and ultimately improve plant growth (Smith and Read, 2008; Han et al., 2022b). Nutrient status, especially of phosphorus, has a strong effect on AM colonization (Nagy et al., 2006; Johnson et al., 2015; Hu et al., 2017b). However, the effect of potassium on AM colonization is more poorly documented. In this study, more than 60% of the roots of *L. barbarum* were colonized by *R. irregularis* (**Table 1**), which was consistent with previous findings (Hu et al., 2017a; Zhang et al., 2017; Han et al., 2022a). Although Garcia et al. (2017) observed that extra potassium has little influence on AM colonization of *Medicago truncatula*, other studies have reported that extra potassium significantly increases the AM colonization of tomato (Liu et al., 2019) and *L. barbarum* (Zhang et al., 2017; Han et al., 2022a; Han et al., 2022b). In the present study, application of potassium increased the AM colonization rate of *L. barbarum* (**Table 1**). This might be due to the imbalance among nitrogen, phosphorus, and potassium, which promotes mycorrhizal development (Bonneau et al., 2013; Zhang et al., 2017). Moreover, the arbuscular rate of *L. barbarum* increased with extra potassium application (**Table 1**), which might reflect that water and nutrient exchange within the arbuscule promotes the development of arbuscular branches (Balestrini et al., 2015).

Overexpression of *LbKAT3* significantly increased the AM colonization arbuscular rate of tobacco under a high potassium concentration (**Figures 7A, B**), and the relative expression of the indicator genes *GintEF1α* and *NtPT4* was consistent with this result (**Figures 7C, D**). These results indicated that improved potassium accumulation can promote AM colonization (Liu et al., 2019). In addition, application of 2 mM potassium significantly increased the AM colonization rate (**Figure 7A**), further indicating that potassium supplementation can promote AM colonization (Zhang et al., 2017; Liu et al., 2019). A high potassium concentration had little influence on the arbuscular rate of OE tobacco (**Figure 6B**), possibly because overexpression of *LbKAT3* had a stronger effect on arbuscular rate than that of potassium supplementation.

### 4.3 Overexpression of *LbKAT3* may promote potassium, phosphorus, and water transport from the AM fungus to the host plant

Inoculation of *R. irregularis* and application of potassium improved the plant phosphorus contents of *L. barbarum* (**Table 2**). Previous studies have similarly observed that the abundance of phosphorus and potassium was correlated in spores (Olsson et al., 2008; Olsson et al., 2011). Moreover, correlation analysis revealed that the root phosphorus content was positively correlated with the potassium content and the expression of *LbKAT3* (**Table 4**). These results suggested that AM fungi and extra potassium are beneficial to phosphorus absorption, which might reflect that potassium is the major counter-ion for polyphosphate, and polyphosphate forms the main phosphate reserve for host plants in the AM symbiosis (Bücking and Heyser, 1999). The gene *PT4* is specifically induced by AM fungi and is involved in phosphorus uptake through the mycorrhizal pathway (Javot et al., 2007; Hu et al., 2017b). The increase in phosphorus content was also associated with upregulation of *LbPT4* expression (**Figure 3A**), and the expression of *LbPT4* was positively correlated with root phosphorus content (**Table 4**). Overexpression of *LbKAT3* increased the expression of *NtPT4* (**Figure 7D**) and phosphorus contents (**Figures 8D–F**) in mycorrhizal tobacco. The root potassium content was positively correlated with the root phosphorus content and expression of *NtPT4* (**Table 5**). In addition, our previous study revealed that, using a three-compartment culture system, foliar-applied potassium in the potassium compartment increased the expression of *LbPT4* of mycorrhizal *L. barbarum* in the potassium-free compartment (Han et al., 2022b). These results may support the hypothesis that the improvement of plant potassium uptake can promote phosphorus uptake by the mycorrhizal symbiosis (Beever and Burns, 1981; Ryan et al., 2003; Garcia et al., 2014), and suggest that overexpression of *LbKAT3* may promote potassium and phosphorus transport from AM fungi to the host plant.

AQPs regulate the water permeability of plasma membranes (Maurel, 1997; Moshelion et al., 2014). *R. irregularis* inoculation and potassium application increased the relative expression of *PIP* and *TIP* genes of *L. barbarum* (**Figures 4A–F**). This result was in accordance with previous findings (Hu et al., 2017a; Coffey et al., 2018), and suggested improvement in root water absorption and transportation, and maintenance of the root cell osmotic balance (Ruiz-Lozano, 2003; Bárzana et al., 2014; Watts-Williams et al., 2019). Previous studies have suggested that extra potassium can improve mycorrhizal root hydraulic conductance to increase water transport by AM fungi, given that the relative expression of fungal *AQP* genes is increased by potassium addition (Kanai et al., 2011; El-Mesbahi et al., 2012). Potassium application upregulated the relative expression of *Rir-AQP1* and *Rir-AQP2* in mycorrhizal *L. barbarum* roots (**Figures 4G, H**). Furthermore, overexpression of *LbKAT3* upregulated the expression of *Rir-AQP1* in mycorrhizal tobacco (**Figure 9A**). Correlation analysis revealed that the root potassium content was positively correlated with the expression of *Rir-AQP1* and *Rir-AQP2* in *L. barbarum* (**Table 4**), and the root potassium content was positively correlated with the expression of *Rir-AQP1* in tobacco (**Table 5**). These results suggested that the increase in potassium uptake was conducive to mycorrhizal water uptake, and overexpression of *LbKAT3* may promote water transport from AM fungi to the host plant.

## 5 Conclusion

Inoculation of *R. irregularis* and application of potassium improved the biomass accumulation, and phosphorus and potassium contents, and upregulated the expression of *LbPT4*, *LbKAT3*, and *Rir-AQP* genes in mycorrhizal *L. barbarum* roots. The LbKAT3 protein is indicated to have potassium uptake ability, and *R. irregularis* upregulated the expression of *LbKAT3* only in AM-colonized roots. Overexpression of *LbKAT3* improved the biomass and potassium and phosphorus contents, and induced the expression of *NtPT4* and *Rir-*AQP genes of mycorrhizal tobacco. *LbKAT3* may assist in mycorrhizal potassium uptake, and overexpression of *LbKAT3* may promote potassium, phosphorus, and water transport from AM fungi to the host plant.

## Data availability statement

The original contributions presented in the study are included in the article/**Supplementary Material**. Further inquiries can be directed to the corresponding authors.

## Author contributions

XH: Conceptualization, Methodology, Investigation, Data curation, Formal analysis, Validation, Visualization, Software, Writing—Original Draft, and Writing—Review and Editing. YZ: Investigation, Methodology, Visualization, and Software. YL: Methodology, Investigation, Visualization, and Software. WR: Methodology, Investigation, Visualization, and Software. KL: Methodology, Investigation, Visualization, and Software. WZ: Methodology and Investigation. HZ: Conceptualization, Writing—Review and Editing, Supervision, Funding acquisition, and Project administration. MT: Supervision, Funding acquisition, and Project administration. All authors contributed to the article and approved the submitted version.
