# Supplementary figures and images for: LbKAT3 may assist in mycorrhizal potassium uptake, and overexpression of LbKAT3 may promote potassium, phosphorus, and water transport from arbuscular mycorrhizal fungi to the host plant
Source: Front Plant Sci. 2023 Jun 20;14:1161220. doi: 10.3389/fpls.2023.1161220 (PMC10319307; doi:10.3389/fpls.2023.1161220)

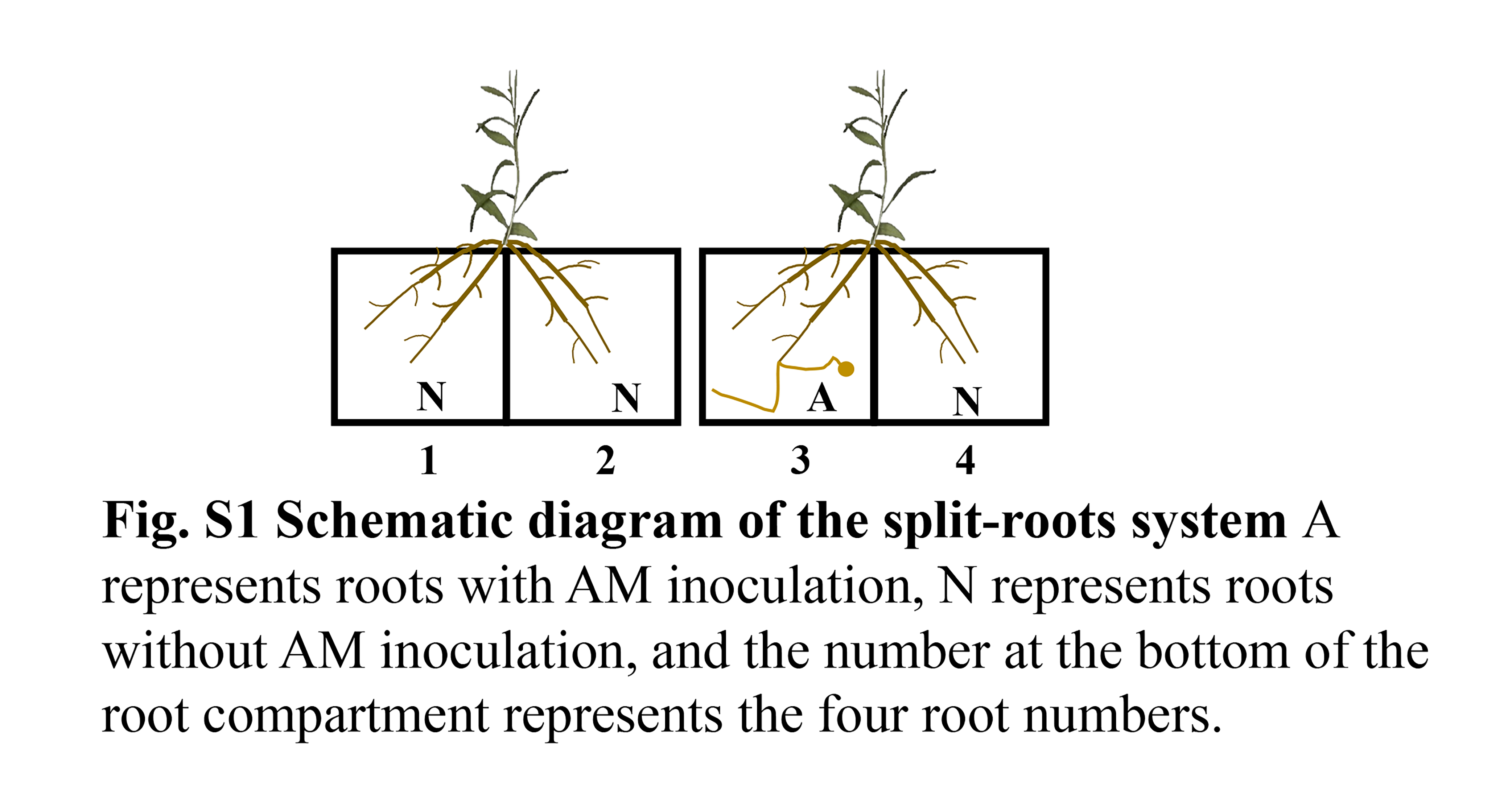

Supplement: Supplementary file 1 [file Image_1.tif]

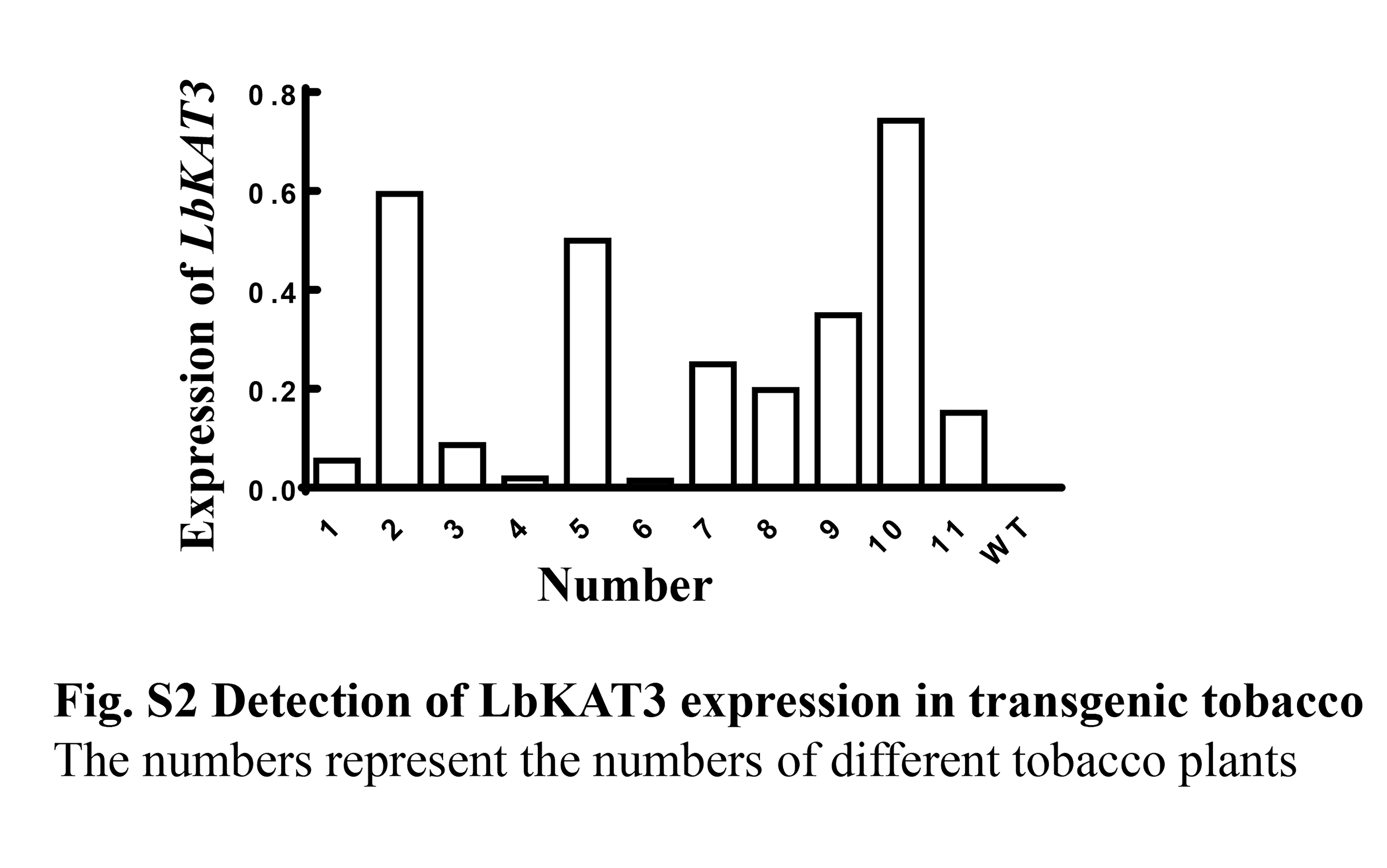

Supplement: Supplementary file 2 [file Image_2.tif]

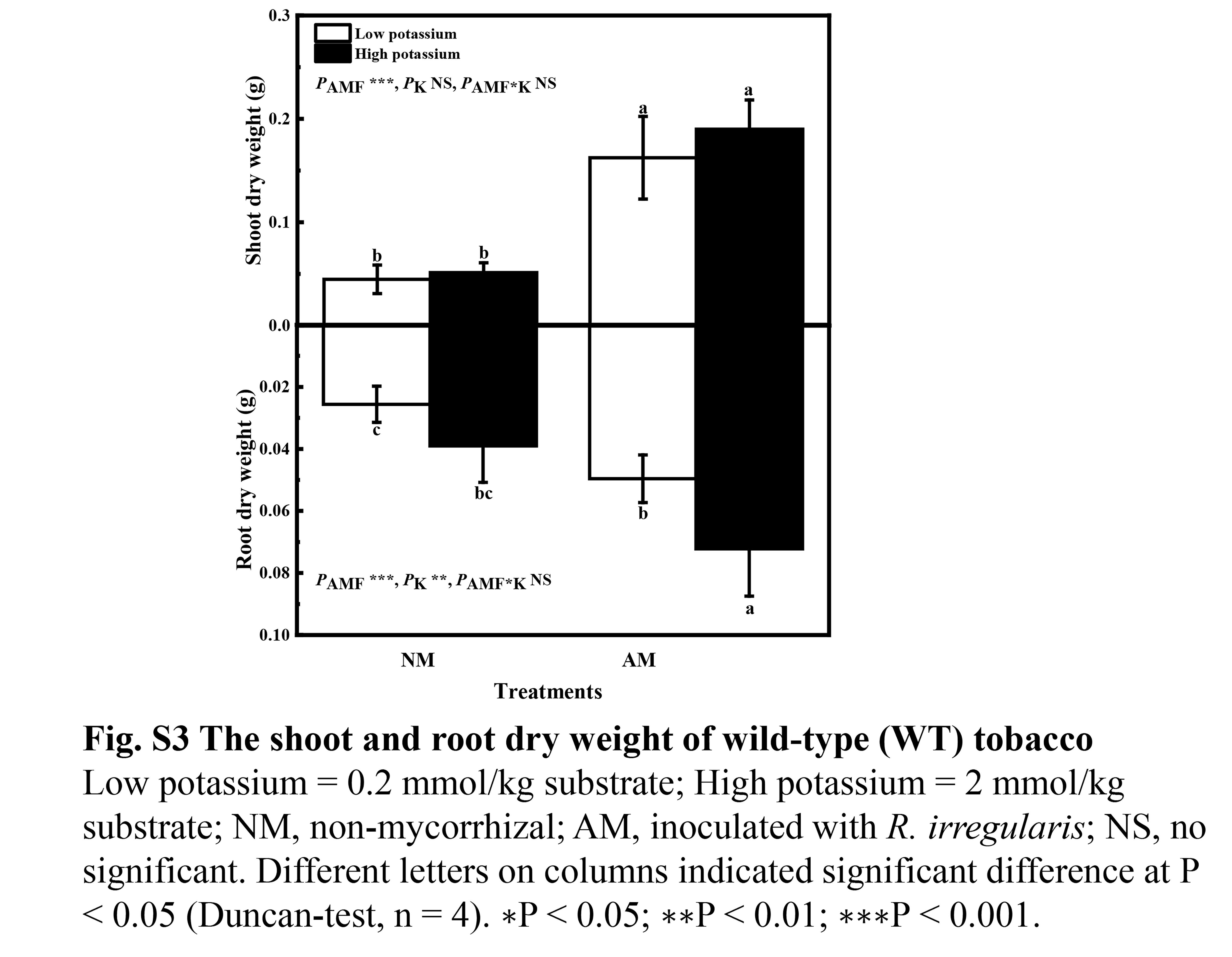

Supplement: Supplementary file 3 [file Image_3.tif]

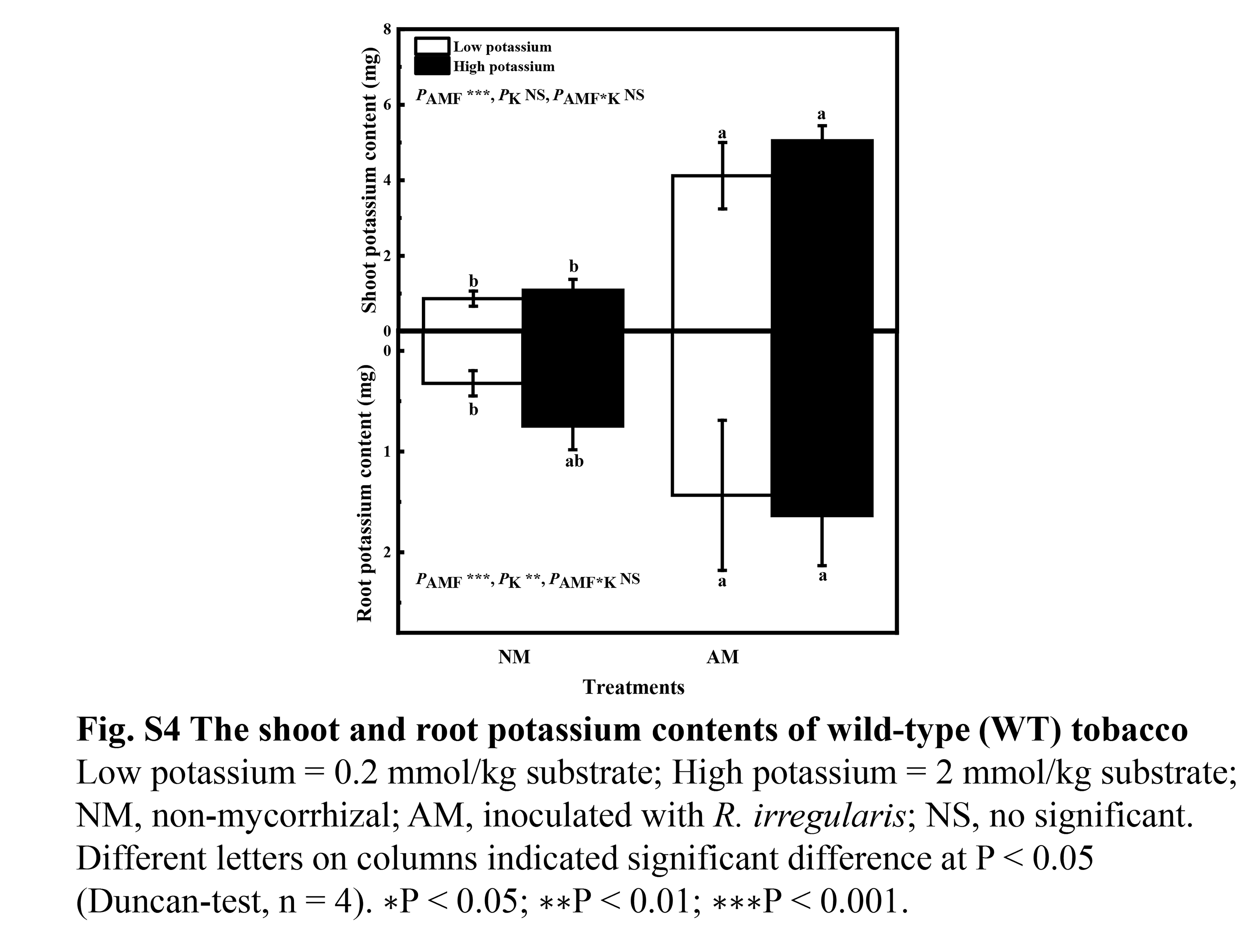

Supplement: Supplementary file 4 [file Image_4.tif]
