# Supplementary material for: LbKAT3 may assist in mycorrhizal potassium uptake, and overexpression of LbKAT3 may promote potassium, phosphorus, and water transport from arbuscular mycorrhizal fungi to the host plant
Source: Front Plant Sci. 2023 Jun 20;14:1161220. doi: 10.3389/fpls.2023.1161220 (PMC10319307; doi:10.3389/fpls.2023.1161220)
Supplement: Supplementary file 5 [file Table_1.docx]

**Supplementary Table 1.** Primers used for clone and quantitative real-time PCR (qRT-PCR)

| Gene name | Primer names | Primer sequences (5’-3’) | Used for |
| --- | --- | --- | --- |
| *LbKAT3* | LbKAT3-3outer | GGGGGACTCTTGCCTGTTGATTTATTG | 3’ RACE |
|  | LbKAT3-3inward | ACTTGCTCAGACTTTGGCGACTACGGCG | 3’ RACE |
|  | LbKAT3-5outer | GGAGCTGCTCAGCGACCTGTATAGTGA | 5’ RACE |
|  | LbKAT3-5inward | GGGGCTGTGGAAGTAGTTACATACGAGA | 5’ RACE |
|  | LbKAT3-f | GAAGACTCCACCCCTATC | Full length PCR |
|  | LbKAT3-r | AGAACATCAAAAGTCGGAA | Full length PCR |
| *LbPT4* | Q-LbPT4-f | TGCATTTGGGATACAACAGTATACGCAAG | qRT-PCR |
|  | Q-LbPT4-r | ATCATTCCCATCCATCATCGTGGT | qRT-PCR |
| *LbKAT3* | Q-LbKAT3-f | TCGGGGAAATAGGTGTGA | qRT-PCR |
|  | Q-LbKAT3-r | TATGAGCGGTATCTGTTGTAG | qRT-PCR |
| *LbKT1* | Q-LbKT1-f | TTCCCAAGATCAACGGGTCATCGG | qRT-PCR |
|  | Q-LbKT1-r | CTTATCACCATCCCGGATTCGAAG | qRT-PCR |
| *LbSKOR* | Q-LbSKOR-f | CTTTTGATATGATTCTTGGTGCTT | qRT-PCR |
|  | Q-LbSKOR-r | CTTTGATATTGTAATCGCAAGTGG | qRT-PCR |
| *LbPIP1-1* | Q-LbPIP1-1-f | GATAAGGATTACAAAGAGCCACC | qRT-PCR |
|  | Q-LbPIP1-1-r | AGCAAAGATCATACCACCAAAAG | qRT-PCR |
| *LbPIP2-1* | Q-LbPIP2-1-f | ATGTTCCCGTATTGGCACCACT | qRT-PCR |
|  | Q-LbPIP2-1-r | GTGTTCATCCCATGCCTTATCACC | qRT-PCR |
| *LbTIP1-1* | Q-LbTIP1-1-f | TATCAGTATGGAACGCCCTTGTGT | qRT-PCR |
|  | Q-LbTIP1-1-r | TTGAAGCTCCAGTAAAGCACCCAC | qRT-PCR |
| *LbTIP2-1* | Q-LbTIP2-1-f | TTGCCATGGATTTGCTCTCTTCGT | qRT-PCR |
|  | Q-LbTIP2-1-r | AGCAATCCAGTAGAACAGGCCCGTA | qRT-PCR |
| *LbTIP3-1* | Q-LbTIP3-1-f | CACGAGCCATAATTTACATAGTTGC | qRT-PCR |
|  | Q-LbTIP3-1-r | ATCATAGGCCATCCAAAGTGACC | qRT-PCR |
| *LbTIP4-1* | Q-LbTIP4-1-f | TTTCAGGAGCTTCAATGAACCC | qRT-PCR |
|  | Q-LbTIP4-1-r | ACAGATAAAGCCAGCAAGACCAC | qRT-PCR |
| *Actin* | Q-Actin-f | TCTACGAGGGTTACGCTTTG | qRT-PCR |
|  | Q-Actin-r | TCCCGTTCAGCAGTGGTT | qRT-PCR |
| *NtPT4* | Q-NtPT4-f | CGCTAGCAAAGCCCAACACAT | qRT-PCR |
|  | Q- NtPT4-r | CGCTAGCAAAGCCCAACACAT | qRT-PCR |
| *NtEF1α* | Q-NtEF1α-f | TATGATTACTGGTACCTCCC | qRT-PCR |
|  | Q-NtEF1α-r | ACCTAGCCTTGGAATACTTG | qRT-PCR |
| *Rir-AQP1* | Q-Rir-AQP1-f | CTTGCTATTCCATTCAGTTTCGG | qRT-PCR |
|  | Q-Rir-AQP1-r | TTTTTTTTACTTTTTTGGGGTCC | qRT-PCR |
| *Rir-AQP2* | Q-Rir-AQP2-f | AGAGTCAGGAGGAGGAACAAGAG | qRT-PCR |
|  | Q-Rir-AQP2-r | CTAACTGCAATACCCAAAGCGAG | qRT-PCR |
| *GintEFα* | Q-GintEFα-f | AATCAGTTGAAATGCACCACGAAC | qRT-PCR |
|  | Q-GintEFα-f | CGACGAATTTCTTTGACTGATACGTT | qRT-PCR |
